# Microbiological, Epidemiological, and Clinical Characteristics and Outcomes of Patients with Cryptococcosis in Taiwan, 1997–2010

**DOI:** 10.1371/journal.pone.0061921

**Published:** 2013-04-17

**Authors:** Hsiang-Kuang Tseng, Chang-Pan Liu, Mao-Wang Ho, Po-Liang Lu, Hsiu-Jung Lo, Yu-Hui Lin, Wen-Long Cho, Yee-Chun Chen, the Taiwan Infectious Diseases Study Network (TIDSnet) for cryptococcosis

**Affiliations:** 1 Institute of Clinical Medicine, National Yang-Ming University, Taipei, Taiwan; 2 Division of Infectious Diseases, Department of Internal Medicine, Mackay Memorial Hospital, Zhongshan District, Taipei, Taiwan; 3 Division of Infectious Diseases, Department of Internal Medicine, China Medical University Hospital, Taichung, Taiwan; 4 Department of Internal Medicine, Kaohsiung Medical University Hospital, Kaohsiung Medical University, Kaohsiung, Taiwan; 5 National Institute of Infectious Diseases and Vaccinology, National Health Research Institutes, Miaoli, Taiwan; 6 School of Dentistry, China Medical University, Taichung, Taiwan; 7 Division of Infectious Diseases, Department of Medicine, Taichung Veterans General Hospital, Taichung, Taiwan; 8 Department of Medicine, Mackay Medical College, New Taipei, Taiwan; 9 Division of Infectious Diseases, Department of Internal Medicine, National Taiwan University Hospital and College of Medicine, Taipei, Taiwan; 10 Mackay Medicine Nursing and Management College, Taipei, Taiwan; California Department of Public Health, United States of America

## Abstract

**Background:**

Among members of *Cryptococcus neoformans*- *Cryptococcus* gattii species complex, *C. neoformans* is distributed worldwide whereas *C. gattii* is considered to be more prevalent in the subtropics and tropics including Taiwan. This nationwide study was undertaken to determine the distribution of genotypes, clinical characteristics and outcomes of 219 patients with proven cryptococcosis at 20 hospitals representative of all geographic areas in Taiwan during 1997–2010.

**Methods and Findings:**

Of 219 isolates analyzed, *C.* neoformans accounted for 210 isolates (95.9%); nine isolates were *C. gattii* (4.1%). The predominant genotype was VNI (206 isolates). The other genotypes included VNII (4 isolates), VGI (3 isolates) and VGII (6 isolates). Antifungal minimal inhibition concentrations higher than epidemiologic cutoff values (ECVs) were found in nine VNI isolates (7 for amphotericin B). HIV infection was the most common underlying condition (54/219, 24.6%). Among HIV-negative patients, liver diseases (HBV carrier or cirrhosis) were common (30.2%) and 15.4% did not have any underlying condition. Meningoencephalitis was the most common presentation (58.9%), followed by pulmonary infection (19.6%) and “others” (predominantly cryptococcemia) (18.7%). The independent risk factors for 10-week mortality, by multivariate analysis, were cirrhosis of liver (P = 0.014) and CSF cryptococcal antigen titer ≥512 (*P* = 0.020). All except one of 54 HIV-infected patients were infected by VNI genotype (98.1%). Of the 13 isolates of genotypes other than VNI, 12 (92.3%) were isolated from HIV-negative patients. HIV-infected patients compared to HIV-negative patients were more likely to have meningoencephalitis and serum cryptococcal antigen ≥1∶512. Patients infected with *C. gattii* compared to *C. neoformans* were younger, more likely to have meningoencephalitis (100% vs. 57%), reside in Central Taiwan (56% vs. 31%), and higher 10-week crude mortality (44.4% vs. 22.2%).

**Conclusions:**

C*ryptococcus neoformans* in Taiwan, more prevalent than C. *gatii*, has a predominant VNI genotype. Isolates with antifungal MIC higher than ECVs were rare.

## Introduction

Among members of the *Cryptococcus neoformans*-Cryptococcus gattii species complex that cause cryptococcosis in humans, *C. neoformans* (comprising var. *grubii* [serotype A] and var. *neoformans* [serotype D]) occur worldwide. In contrast, *C. gattii* (serotype B and C) is usually limited to the selected regions, particularly the Asia-Pacific region before the occurrence of a *C. gattii* outbreak in Vancouver Island, Canada [1]. Based on a large global molecular epidemiologic survey *Cryptococcus* could be divided into eight major genotypes: VNI (serotype A), VNII (serotype A), VNIII (serotype AD), and VNIV (serotype D) of C. neoformans; and VGI, VGII, VGIII, and VGIV of *C. gattii* using orotidine monophosphate pyrophosphorylase (*URA5*) gene restriction fragment length polymorphism (RFLP) analysis and M13 polymerase chain reaction (PCR) fingerprinting [2].

Cryptococcosis is associated with significant morbidity and mortality. It can present as meningoencephalitis, pneumonia and cryptococcemia in both immunocompetent and immunocompromised hosts. Outcome and treatment failure are usually associated with underlying conditions, a delay in diagnosis, and absence of a fungicidal drug [3]–[5]. In addition, the emergence of isolates with resistance or elevated minimum inhibition concentration (MIC) above epidemiologic cutoff values (ECVs) is of concern as well [6], [7].

We conducted this nationwide multicenter retrospective study for patients with proven cryptococcosis to address two questions. First, what are the genotypes and antifungal susceptibility of C*ryptococcus* clinical isolates collected from representative regions in Taiwan? Second, are demographic qualities, underlying conditions, and microbiological characteristics associated with cryptococcosis patient mortality?

## Population and Methods

This research was approved by the Research Ethics Committees of the National Taiwan University Hospital (No. 201209035RIC), Mackay Memorial Hospital (No.12MMHIS120), Kaohsiung Medical University Hospital (No.KMUH-IRB-20120239), China Medical University Hospital (No. DMR101-IRB1-240), and National Health Research Institute (No.EC 09602024) and was conducted according to the Declaration of Helsinki. The project involved the use of existing data, records, and clinical isolates without intervention. Informed consent was waived and the data were analyzed anonymously.

### Hospital settings and *Cryptococcus* clinical isolates

*Cryptococcus* clinical isolates were obtained from 219 patients with proven cryptococcosis managed at 20 hospitals located in the four geographic regions of Taiwan during 1997–2010. The initial patient isolate, regardless of anatomical site, was selected and sent to National Taiwan University Hospital (NTUH) for microbiological characterization.

### Genotypes

High-molecular-weight DNA was isolated and genotypes were determined by *URA5* gene RFLP analysis [2]. Molecular types were evaluated and compared using M13 PCR-fingerprinting [2]. The computer program BioNumerics version 6.0 (Applied Maths, Kortrijk, Belgium) was used to determine the cluster analysis by the UPGMA method [8]. DNA bands were defined manually with a band position tolerance of 0.8% and an optimization setting of 0.2%. Reference strains included WM 148 (VNI), WM 626 (VNII), WM 628 (VNIII), WM 629 (VNIV), WM 179 (VGI), WM 178 (VGII), WM 161 (VGIII), WM 779 (VGIV) [2], two Australia clinical strains T184 (VNI) and T185 (VGI), and Vancouver Island outbreak strains R265 (VGIIa) and R272 (VGIIb).

### Antifungal susceptibility

Susceptibility, as displayed by MIC (µg/ml) levels, to amphotericin B, flucytosine, fluconazole, and voriconazole was determined following the Clinical Laboratory Standards Institute (CLSI) M27-A3 broth microdilution method and incubated at 35°C [9]. All results were read visually at 72 h. The reference strains *C. neoformans* ATCC 90112, *Candida albicans* ATCC 90028, and *Candida parapsilosis* ATCC 22019 were used as internal controls. The ECVs are the MIC values that captured >95% of the observed population in RPMI medium provided in recent studies [6], [7].

### Clinical characteristics and outcomes of patients with cryptococcosis

Data were collected retrospectively after isolates were sent for microbiological characterization and included gender, age, underlying conditions such as human immunodeficiency virus (HIV) status and lowest CD4 count during hospitalization, hepatitis B virus (HBV) carrier defined by positive surface antigen (HBsAg) status, and cirrhosis of liver determined by sonography; clinical characteristics included presentation, initial cryptococcal capsular polysaccharide antigen titer in cerebrospinal fluid (CSF) or serum, baseline intracranial opening pressures, neurosurgical intervention, all-cause mortality at 2- and 10-weeks. One patient could possess more than one underlying condition. We did not collect and record treatment details.

### Case definition

Proven cryptococcosis was defined and classified into cryptococcal meningoencephalitis, pulmonary cryptococcosis, and others as described previously [10].

### Data analysis

The categorical variables were analyzed by number (No.) (%) and the continuous variables were presented as mean ± standard deviation (SD). The association between categorical variables was analyzed with the Chi-square test or Fisher's exact test if the expected number was less than five. The independent and joint effects of several variables to identify significant predictors of mortality were investigated by univariate and multivariate logistic regression analyses. Two-sided P value <0.05 was considered statistically significant. All statistical analyses were performed using the SAS software, version 9.2 (SAS Institute Inc., Cary, NC, US).

## Results

### *Cryptococcus* genotypes

Of 219 *Cryptococcus* clinical isolates, 210 were *C. neoformans* (95.9%) and 9 were *C. gattii* (4.1%). VNI genotype accounted for 206/210 (98.1%) of C. neoformans. Four isolates were VNII. Among the nine isolates of *C. gattii*, three were VGI and six were VGII. The details of patients with VNII and *C. gattii* are shown in **Table S1** and **Table S2**, respectively.

**Figure 1:**
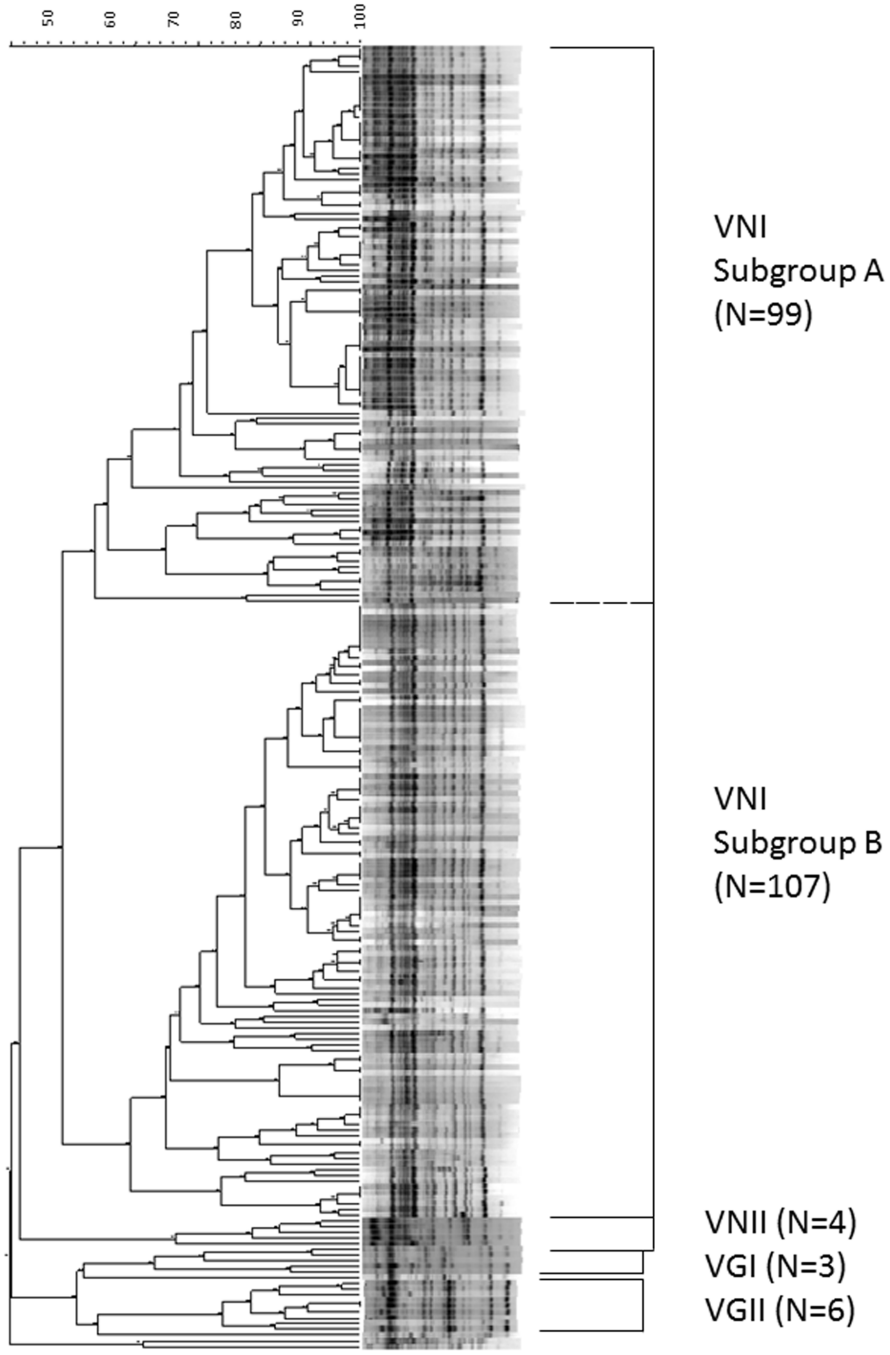
Dendrogram of M13 PCR fingerprint analysis of 219 clinical isolates of *Cryptococcus neoformans*-*Cryptococcus* gattii species complex collected in Taiwan during 1997 to 2010 and 12 reference strains.

**Figure 1** shows the M13 PCR-fingerprinting dendrogram of the 219 cryptococcal isolates (details are presented in **Figure S1**). Genotype VNI can be divided into two subgroups. Subgroup A accounted for 48.1% (99/206) of VNI with 57.4% similarity and subgroup B accounted for 51.9% (107/206) of VNI with 63.2% similarity.

### Antifungal susceptibility

Among the 219 isolates, the susceptibility data of three VNI isolates (T203, T205, and T262) were indeterminate due to very poor growth in RPMI broth at 35°C. The MIC levels of 216 isolates to amphotericin B, flucytosine, fluconazole, and voriconazole are shown in **Table 1**. Seven of 203 VNI isolates (3.4%) had amphotericin B MIC levels higher than ECV. One VNI isolate had a flucytosine MIC level higher than ECV. Two of six VGII isolates and one of 203 VNI isolates had fluconazole MIC levels >8 µg/ml, but there were none above this level for 4 VNII isolates and 3 VGI isolates. Fluconazole ECV was 8 µg/ml for VNI and VGI, and was 32 µg/ml for VGII. Therefore, only one VNI isolate of 219 isolates had fluconazole MIC higher than ECV. Detailed information regarding cryptococcosis due to *Cryptococcus* VNI isolates with antifungal MICs higher than ECVs is shown in **Table S3**.

**Table 1:** Susceptibility of 216 cryptococcal clinical isolates to four antifungal agents in Taiwan, 1997–2010.

Antifungal agent	Genotype	No. of isolates	Minimum inhibitory concentration (µg/mL)					% (No.) above ECV	
			Range	Geometric Mean	MIC_50_	MIC_90_	ECV	This study	Global studies^a^
**Amphotericin B**									
	VNI	203	0.03–1	0.48	0.5	0.5	0.5	3.4% (7)	2.8%
	VNII	4	0.13–1	0.42	0.5	1	NA^a^		
	VGI	3	0.25–0.25	0.25	0.25	0.25	0.5	0%	0.8%
	VGII	6	0.06–1	0.31	0.5	1	1	0%	0.8%
**Flucytosine**									
	VNI	203	0.13–32	1.14	1	2	8	0.5% (1)	3.4%
	VNII	4	0.13–2	0.30	0.19	2	NA^a^		
	VGI	3	0.5–1	0.63	0.5	1	4	0%	4.3%
	VGII	6	1–2	1.59	2	2	16	0%	2.9%
**Fluconazole**									
	VNI	203	0.03–16	2.35	4	8	8	0.5% (1)	2.9%
	VNII	4	0.13–8	0.84	0.75	8	NA^a^		
	VGI	3	1–4	2	2	4	8	0%	1.2%
	VGII	6	0.13–16	5.04	8	16	32	0%	6.9%
**Voriconazole**									
	VNI	203	0.03–0.25	0.06	0.06	0.13	0.25	0%	2.4%
	VNII	4	0.03–0.13	0.05	0.05	0.13	NA^a^		
	VGI	3	0.03–0.06	0.04	0.03	0.06	0.5	0%	0%
	VGII	6	0.13–0.25	0.20	0.25	0.25	0.25	0%	4.1%

a The epidemiologic cutoff values of VNII to antifungal drugs being tested were not available in global studies [6], [7].

### Epidemiological and clinical characteristics

**Table 2** shows the epidemiological and clinical characteristics of the 219 patients with proven cryptococcosis. More than half of the patients were in Northern Taiwan. However, 5 of 9 isolates of *C. gattii* (55.6%) were from Central Taiwan. The most common five underlying conditions were HIV infection (54 patients, 24.6%), HBV carrier (46 patients, 21.0%), malignancies (44 patients, 20.1%), diabetes mellitus (40 patients, 18.2%), and cirrhosis of liver (31 patients, 14.1%). No underlying condition was identified in 23 patients (10.5%). Meningoencephalitis was the most common presentation (58.9%), followed by pulmonary infection (19.6%) and “others” (predominantly cryptococcemia) (18.7%). The nine patients with *C. gattii* infection, compared to 210 patients with C. neoformans, were younger (mean 38.6 years vs. 53.1 years) and more likely to have no underlying conditions (44.4% vs. 9.0%), to have meningoencephalitis (100.0% vs. 57.1%) and to undergo neurosurgical intervention (33.3% vs. 9.0%). They also had a higher 10-week mortality (44.4% vs. 22.2%), as seen in **Table 2**.

**Table 2:** Epidemiological and clinical characteristics of 219 patients with proven cryptococcosis hospitalized at 20 hospitals in Taiwan, 1997–2010.

Characteristics	*Cryptococcus neoformans* (N = 210)		*Cryptococcus gattii* (N = 9)	
	No.	(%)	No.	(%)
Geographic distribution				
Northern	119	(56.7)	1	(11.1)
Central	65	(30.9)	5	(55.6)
Southern	21	(10.0)	1	(11.1)
Eastern	5	(2.4)	2	(22.2)
Demographic data				
Age, range, years	12 to 94		22 to 68	
Age, mean ± SD, years	53.1±18.4		38.6±13.0	
Age ≥60 years	75	(35.7)	1	(11.1)
Male	143	(72.2)	5	(55.6)
Underlying conditions				
HIV infection	53	(27.3)	1	(11.1)
Liver diseases				
Hepatitis B virus carrier	46	(21.9)	0	(0.0)
Cirrhosis of liver	31	(14.8)	0	(0.0)
Malignancy				
Hematological malignancy	13	(6.2)	0	(0.0)
Other malignancy	31	(14.8)	0	(0.0)
Diabetes mellitus	39	(18.6)	1	(11.1)
Kidney diseases	21	(9.6)	0	(0.0)
Systemic lupus erythematosus and other rheumatologic diseases	11	(5.2)	0	(0.0)
Cerebrovascular accident	8	(3.8)	1	(11.1)
Tuberculosis	6	(2.9)	0	(0.0)
Solid organ transplantation^a^	3	(1.4)	1	(11.1)
Idiopathic CD4 lymphocytopenia	3	(1.4)	0	(0.0)
Other diseases	3	(1.4)	0	(0.0)
No underlying conditions	19	(9.0)	4	(44.4)
Classification of cryptococcosis				
Meningoencephalitis	120	(57.1)	9	(100.0)
Pulmonary cryptococcosis	43	(20.5)	0	(0.0)
Others^b^	47	(22.4)	0	(0.0)
Serum cryptococcal capsular antigen				
Antigen titer ≥512	73	(34.8)	4	(44.4)
Antigen titer <512	57	(27.1)	3	(33.3)
Not done	80	(38.1)	2	(22.2)
CSF cryptococcal capsular antigen				
Antigen titer ≥1∶512	76	(36.2)	7	(77.8)
Antigen titer <1∶512	40	(19.0)	2	(22.2)
Not done	94	(44.8)	0	(0.0)
Intracranial pressure				
Opening pressure≥250 mmH_2_O	48	(22.9)	6	(66.7)
Opening pressure<250 mmH_2_O	42	(20.0)	2	(22.2)
Not done or not available	120	(57.1)	1	(11.1)
Neurosurgical intervention	19	(9.0)	3	(33.3)
All-cause mortality				
2-week mortality	22	(10.5)	2	(22.2)
10-week mortality	60	(28.6)	4	(44.4)

Abbreviations: SD: standard deviation; CSF: cerebrospinal fluid; HIV: human immunodeficiency virus.

a Solid organ transplantation included two liver transplantations and one heart transplantation in *C. neoformans* infected patients; and one kidney transplantation in *C. gattii* infected patient.

b “Others” included 36 patients with cryptococcemia.

Of 54 HIV-infected patients, 53 were infected by the VNI genotype (98.1%) and one was infected by the VGI genotype, as seen in **Table 3**. Excluding five patients without recorded CD4 data, the mean CD4 of 49 HIV-infected patients was 50.0±68.3/mL (ranging from 2 to 318/mL). Of 13 isolates of genotypes other than VNI, twelve (92.3%) were isolated from HIV-negative patients (**Table 3** **, Table S1, and Table S2**). The 54 HIV-infected patients, as compared to the 149 HIV-negative patients, were younger, predominantly male, and more likely to have meningoencephalitis and serum cryptococcal antigen ≥512. Compared to HIV infected patients, HIV-negative patients were more likely to have pulmonary infection and liver diseases (either HBV carrier or cirrhosis of liver) as the most common underlying conditions (45 patients, 30.2%).

**Table 3:** Comparisons of genotype distribution and clinical characteristics of cryptococcosis by HIV status, Taiwan, 1997–2010.

Characteristics	HIV-negative patients (N = 149)^a^		HIV-infected patients (N = 54)^a^		*P* value
	No.	(%)	No.	(%)	
Genotype distribution					
VNI	137		53		
VNII	4		0		
VGI	2		1		
VGII	6		0		
Geographic distribution					
Northern	84	(56.4)	34	(63.0)	
Central	43	(28.9)	14	(25.9)	
Southern	16	(10.7)	5	(9.3)	
Eastern	6	(4.0)	0	(0.0)	
Demographic data					
Age ≥60 years	75	(50.3)	1	(1.9)	<0.001
Male	94	(63.1)	51	(94.4)	<0.001
Underlying conditions					
Liver diseases					
Hepatitis B virus carrier	33	(22.1)	13	(24.1)	0.845
Cirrhosis of liver^b^	30	(20.1)	1	(1.9)	0.001
Diabetes mellitus	40	(26.8)	0	(0.0)	<0.001
Malignancy					
Hematological malignancy	5	(3.4)	3	(5.6)	0.686
Other malignancy	33	(22.1)	3	(5.6)	0.005
Kidney diseases	20	(13.4)	1	(1.9)	0.014
Solid organ transplantation	4	(2.7)	0	(0.0)	0.576
No underlying conditions	23	(15.4)	0	(0.0)	0.002
Classification of cryptococcosis					0.002
Meningoencephalitis	80	(53.7)	44	(81.5)	
Pulmonary cryptococcosis	35	(23.5)	3	(5.6)	
Others^c^	34	(22.8)	7	(13.0)	
Serum cryptococcal capsular antigen					0.001
Antigen titer ≥512	43	(28.9)	34	(63.0)	
Antigen titer <512	49	(32.9)	11	(20.4)	
Not done^d^	57	(38.3)	9	(16.7)	
CSF c cryptococcal capsular antigen					0.661
Antigen titer ≥1∶512	50	(33.6)	33	(61.1)	
Antigen titer <1∶512	27	(18.1)	15	(27.8)	
Not done^d^	72	(48.3)	6	(11.1)	
Intracranial pressure					0.101
Opening pressure≥250 mmH_2_O	32	(21.5)	22	(40.7)	
Opening pressure<250 mmH_2_O	33	(22.1)	11	(20.4)	
Not done or not available^d^	84	(56.4)	21	(38.9)	
Neurosurgical intervention	15	(10.1)	7	(13.0)	0.592
All-cause mortality					
2-week mortality	19	(12.8)	5	(9.3)	0.468
10-week mortality	52	(34.9)	12	(22.2)	0.100

Abbreviation: HIV: human immunodeficiency virus.

a Of 219 patients with cryptococcosis, the HIV status of 16 patients was not available. Therefore, 203 cases were included for analysis.

b One patient could possess more than one underlying condition; 18 HIV-negative patients had both cirrhosis of liver and HBV infection.

c “Others” included 25 patients with cryptococcemia in HIV-negative group and seven cryptococcemia in HIV-infected group.

d Data which were not done or not available were excluded from statistical analysis.

Of nine patients infected by the VNI genotype and with antifungal MICs above ECVs, five patients had HIV infections, six had meningoencephalitis, and three had cryptococcemia. The all-cause mortality at 10 weeks was 33.3% (3/9), as shown in **Table S3**. We did not collect data, such as prior use of antifungal agent or drug interaction, to explain the reason for elevated MICs.

### Risk factors for mortality at 2 weeks and 10 weeks

The outcomes of 19 patients at 2-weeks and 24 patients at 10-weeks were not available as patients transferred to other hospitals. All-cause mortality at 2-weeks and 10-weeks were shown in **Table 1**. The significant risk factors for 2-week mortality of cryptococcosis, according to univariate analysis, were geographic distribution in Eastern Taiwan (P = 0.041), and classification of “others” (predominantly cryptococcemia) (P = 0.011). Under multivariate analysis the risk factors for 2-week mortality were geographic distribution in Eastern Taiwan (P = 0.043; odds ratio (OR), 10.7; 95% confidence interval (CI), 1.1–106.1) and classification of “others” (P = 0.018; OR, 13.3; 95% CI, 1.6–112.4).

Risk factors associated with 10-week mortality for 195 patients with cryptococcosis are shown in **Table 4**. The significant factors under univariate analysis were age ≥60 years (P = 0.016), cirrhosis of liver (P = 0.001), kidney diseases (P = 0.035), meningoencephalitis (P = 0.038), other cryptococcosis (P<0.001) and CSF cryptococcal antigen titer ≥1∶512 (P = 0.019). Multivariate analysis showed cirrhosis of liver (P = 0.014; OR, 3.8; 95% CI, 1.3–11.16) and CSF antigen titer ≥1∶512 (P = 0.020; OR, 3.3; 95% CI, 1.2–9.0) as independent predictors for mortality.

**Table 4:** Risk factors associated with 10-week mortality for 195 patients with cryptococcosis in Taiwan.

Characteristics	Died (N = 64)		Lived (N = 131)		Odds ratio	95% confidence interval	*P* value
	No.	(%)	No.	(%)			
Demographic data							
Age ≥60 years	32	(50.0)	42	(32.1)	2.2	1.1–3.9	0.016
Male	41	(64.1)	98	(74.8)	0.6	0.3–1.1	0.12
Underlying conditions							
HIV infection	12	(18.8)	39	(29.8)	0.5	0.3–1.1	0.10
Hepatitis B virus carrier	15	(23.4)	28	(21.4)	1.1	0.5–2.3	0.76
Cirrhosis of liver	18	(28.1)	12	(9.2)	3.9	1.7–8.7	0.001
Kidney diseases	11	(17.2)	9	(6.9)	2.7	1.1–7.0	0.03
Classification of cryptococcosis							
Pulmonary	5	(7.8)	33	(25.2)	1.0		
Meningoencephalitis	37	(57.8)	83	(63.4)	2.9	1.1–8.1	0.04
Others^a^	22	(34.4)	15	(11.4)	10.4	3.3–32.9	<0.001
Serum cryptococcal capsular antigen							
Antigen titer ≥1∶512	26	(40.6)	47	(35.9)	1.4	0.7–2.9	0.41
Antigen titer <1∶512	17	(26.6)	42	(32.1)	1.0		
Not done^b^	21	(32.8)	42	(32.1)			
CSF cryptococcal capsular antigen							
Antigen titer ≥1∶512	29	(45.3)	51	(38.9)	3.2	1.2–8.6	0.02
Antigen titer <1∶512	6	(9.4)	34	(26.0)	1.0		
Not done^b^	29	(45.3)	46	(35.1)			
Intracranial pressure							
Opening pressure ≥250 mmH_2_O	16	(25.0)	37	(28.2)	1.0	0.4–2.6	0.92
Opening pressure <250 mmH_2_O	12	(18.8)	29	(22.1)	1.0		
Not done or not available^b^	36	(56.3)	65	(49.6)			
Neurosurgical intervention	9	(14.1)	13	(9.9)	1.5	0.6–3.7	0.43

Abbreviation: CSF: cerebrospinal fluid.

a “Others” included 19 patients with cryptococcemia died and 12 patients with cryptococcemia lived.

b Data which were not done or not available were excluded from statistical analysis.

## Discussion

The current study provides the first nationwide description of the microbiological and clinical epidemiology of cryptococcosis in Taiwan. The majority of isolates in Taiwan were *C. neoformans* genotype VNI (96%). This is in agreement with the worldwide distribution of *Cryptococcus* which is VNI in Ibero-America (68%) [2], Vietnam (71%) [11], India (89%) [12], Malaysia (89%) [13], China (93%) [14] and Korea (96%) [15].

Cryptococcosis in HIV-negative patients was common (73%) in Taiwan (this study) as well as in China (84% to 96%) [14], [16], [17]. However, HIV-negative patients accounted for 60% in an Indian study [12], 57% in Australia and New Zealand [18], 23% of a French cohort [19] and 18% in Mexican [20]. Only 15% patients were no underlying condition in Taiwan (this study). This was very different from reports in China (68%) [16] and Vietnam (81%) [11]; and yet was close to a study in Korea (19%) [15], USA (22%) [10] and results of another review from China (16%) [17]. Regarding the distribution of underlying conditions and their impact on 10-week mortality, this study showed that HIV infection was the most common underlying condition (25%), but not a risk factor associated with mortality of cryptococcosis (**Table 4**). Liver diseases (either HBV carrier or cirrhosis) were the most common underlying conditions among HIV-negative patients in Taiwan (30%, **Table 3**) and in China (12%) [17]. Furthermore, cirrhosis of liver was an independent predictor of mortality in this study (**Table 4**) and our previous single center study of cryptococcemia [21]. High CSF antigen titers have been associated with death at 10 weeks in a cohort of Italian HIV-positive patients [22] and HIV uninfected patients in Vietnam [11] and our previous study [23]. Our current study confirmed this finding as well. Thus, a threshold of 1∶512 or higher should help monitor patients with cryptococcosis, regardless of their HIV status.

In this study, we found clinical presentation of patients with *C. gattii* infection were more likely than those with *C. neoformans* infection to have meningoencephalitis, were younger, and were less likely to have underlying conditions (**Table 2**), which was concordant with an Australian study [18]. The past studies from a center in northern Taiwan (i.e. NTUH) revealed that clinical cases of *C. gattii* decreased from 59% (17/29) during 1982–1994 to 13% (4/30) during 1995–1997 [24], and 1% (1/100) during 1999–2004 [25]. Another report from a center in southern Taiwan showed 15% (5/34) clinical cases during 1998–2002 were *C. gattii* [26]. Although the ecological niches of *C. gattii* are poorly defined in Taiwan [27], Chaturvedi V. et al. suggested a hypothetical lifecycle of *C. gattii* whereby it cycles through plants, soil, air, and water [28]. Loss of tree coverage in mountainous areas following numerous landslides washed into the estuaries in recent years might explain part of the reason why there has been a decrease in *C. gattii* in Taiwan. We speculate that the global distribution of *C. gattii*, as shown in **Table 5**, might be related to ocean circulation to allow distribution and thriving of *C. gattii* propagules into new ecological niches.

**Table 5:** The global distribution of clinical isolates of *Cryptococcus* gattii by genotype in the literature reviewed.

Report year	Collection year	Region	No. of isolates					Reference
			Total	VGI	VGII	VGIII	VGIV	
1996	1965–1994	Australia	48	44	3	1	0	[33]
2003	1961–2001	South American	33	3	13	16	1	[2]
2004	1999–2002	Canada, BC	21	1	20	0	0	[8]
2005	NA	Papua New Guinea	37	31	2	4	0	[34]
2005	NA	Australia, NT	21	9	12	0	0	[34]
2005	NA	India	5	0	5	0	0	[12]
2006	1987–2004	Colombia	16	1^a^	14^b^	1	0	[35]
2006	1998–2003	Hong Kong	3	1	2	0	0	[36]
2007	2004–2005	USA, Northwest	5	1	4	0	0	[37]
2008	1994–2006	China, 16 provinces	9	9	0	0	0	[16]
2008	1981–2005	China, Southeastern	9	8	1	0	0	[14]
2009	2006–2008	USA, Northwest	14	0	14	0	0	[38]
2009	1994–2004	Mexico	8	2	2	2	2	[20]
2009	2007	USA, Southeastern	1	1	0	0	0	[39]
2010	2003–2004	Malaysia	11^c^	4	4	0	0	[13]
2010	1998–2007	Vietnam	10	9	1	0	0	[11]
2010	1990–2008	Korea	2	0	1	1	0	[15]
2010	2007	Japan	1	0	1	0	0	[40]
2012	2005–2007	India	4	0	0	0	4	[41]
2012	2011	USA, Southeastern	1	1	0	0	0	[42]
2012	1997–2010	Taiwan	9	3	6	0	0	Current Study

Abbreviations: NT: Northern Territory; BC: British Columbia; NA: not available.

a Mating type **a**.

b 11 strains with mating type **a** were included.

c Three untyped *C. gattii* were included.

Recently, Espinel-Ingroff A. et al. suggested the epidemiologic cutoff values (ECVs) (highest wild type susceptibility endpoint) of antifungal susceptibility for reference [6], [7] as the Clinical and Laboratory Standards Institute (CLSI) does not provide clinical breakpoints (CBPs) for *Cryptococcus* species [9]. While CBPs predict the clinical outcome of therapy, the ECVs could monitor the emergence of strains with reduced susceptibility (due to mutation) to the agent being evaluated. In the current study, only nine of 219 isolates had MICs higher than ECVs (**Table 1**). Of them, seven isolates (3.4%) of the VNI genotype had amphotericin B MIC levels higher than ECV, while the global study showed 2.8% [6]. Regarding fluconazole MIC, the values of MIC_50_ and MIC_90_ in this study (**Table 1**) and ECVs in global studies [7] were higher for VGII than for VGI, VNI, and VNII. This indicates antifungal susceptibility for *Cryptococcus* should be species-specific and molecular type-specific [6], [7]. It seems likely that the differences seen among the *C. neoformans*- *C. gattii* species complex are due to intrinsic heteroresistance to fluconazole [29], chromosome duplication during prolonged azole therapy [30], and possible involvement of phosphoinositide-dependent kinase (PDK1), protein kinase C (PKC), and target of rapamycin (TOR) signaling pathways in basal fluconazole tolerance [31].

The strengths of this study are the large number of cryptococcal clinical isolates collected from hospitals representative of all regions of Taiwan during a 13 year period, the use of molecular methods for genotyping, assessment of antifungal susceptibility, and characterization of the risk factors for 10-week mortality. The weaknesses inherent in a study of this kind were the inability to collect sufficient isolates of rare genotypes or those with MICs higher than ECV to determine the impact on outcome. Generally only one isolate per infection is tested, although it has been revealed that 20% of patients with cryptococcosis can be infected by multiple strains or molecular types [32].The geographic distribution according to hospital location might not represent the places where exposure to *Cryptococcus* occurred. Besides, we could not evaluate treatment responses of an individual drug because antifungal regimens and dosages were modified in many of the patients and confounded by the underlying conditions.

In conclusion, the major genotype of *Cryptococcus* clinical isolates in Taiwan was VNI. Only nine of 219 patients were infected by *C. gattii*. Isolates with antifungal MICs higher than ECVs were rare. HIV infection was the most common underlying condition and all except one such patient was infected by the VNI genotype. Liver diseases were the most common underlying conditions in HIV-negative patients. Cirrhosis of liver and high CSF cryptococcal antigen levels were independent predictors of 10-week mortality.

## Supporting Information

Figure S1 Details of dendrogram of M13 PCR fingerprint analysis of 219 clinical isolates of *Cryptococcus neoformans*- *Cryptococcus* gattii species complex collected in Taiwan during 1997 to 2010 and 12 reference strains.
(TIF)

Table S1 Microbiological, epidemiological, and clinical characteristics and outcomes of cryptococcosis due to VNII genotype in Taiwan, 1997 to 2010.
(DOC)

Table S2 Microbiological, epidemiological, and clinical characteristics and outcomes of *Cryptococcus* gattii in Taiwan, 1997 to 2010.
(DOC)

Table S3 Microbiological, epidemiological, and clinical characteristics and outcomes of cryptococcosis due to *Cryptococcus* VNI isolates with antifungal minimum inhibition concentration above epidemiologic cutoff values in Taiwan, 1997 to 2010.
(DOC)
