# Supplementary material for: Microbiological, Epidemiological, and Clinical Characteristics and Outcomes of Patients with Cryptococcosis in Taiwan, 1997–2010
Source: PLoS One. 2013 Apr 17;8(4):e61921. doi: 10.1371/journal.pone.0061921 (PMC3629109; doi:10.1371/journal.pone.0061921)
Supplement: Table S1 — Microbiological, epidemiological, and clinical characteristics and outcomes of cryptococcosis due to VNII genotype in Taiwan, 1997 to 2010. (DOC) [file pone.0061921.s002.doc]

**Table S1. Microbiological, epidemiological, and clinical characteristics and outcomes of cryptococcosis due to VNII genotype in Taiwan, 1997 to 2010**

| No. | Age | Sex | Year | Month | Geographic distribution | Isolate No. | Specimen | AMB MIC | FCT MIC | FLU MIC | VOR MIC | Classification of cryptococcosis | Underlying conditions | Antigen titer ≥1:512 | Intracranial pressure ≥250 cmH_2_O | Neuro surgical intervention | Outcomes |
| --- | --- | --- | --- | --- | --- | --- | --- | --- | --- | --- | --- | --- | --- | --- | --- | --- | --- |
| 1 | 83 | F | 2003 | 3 | Northern Taiwan | T298 | Sputum | 0.13 | 2 | 8 | 0.13 | Pulmonary | Hepatitis B virus carrier, Cerebrovascular accident | No | NA | Not done | Died at 10 weeks |
| 2 | 46 | M | 2006 | 7 | Eastern Taiwan | T167 | CSF | 1 | 0.25 | 0.5 | 0.03 | Meningoencephalitis | Cirrhosis of liver, Hepatitis B virus carrier, Diabetes mellitus, Kidney disease | Serum, CSF | NA | Not done | Survive |
| 3 | 67 | M | 2009 | 5 | Northern Taiwan | T145 | CSF | 0.5 | <0.13 | <0.13 | 0.03 | Meningoencephalitis | Tuberculosis, Lung cancer | No | NA | Not done | Survive |
| 4 | 42 | F | 2009 | 6 | Northern Taiwan | T173 | CSF | 0.5 | 0.13 | 1 | 0.06 | Meningoencephalitis | Cirrhosis of liver | Serum, CSF | NA | Not done | Survive |

Abbreviations: M: male, F: female; CSF: cerebrospinal fluid; AMB: amphotericin B, FCT: flucytosine, FLU: fluconazole, VOR: voriconazole, MIC: minimal inhibition concentration (µg/ml); NA: not available.
