# Supplementary material for: Microbiological, Epidemiological, and Clinical Characteristics and Outcomes of Patients with Cryptococcosis in Taiwan, 1997–2010
Source: PLoS One. 2013 Apr 17;8(4):e61921. doi: 10.1371/journal.pone.0061921 (PMC3629109; doi:10.1371/journal.pone.0061921)
Supplement: Table S2 — Microbiological, epidemiological, and clinical characteristics and outcomes of Cryptococcus gattii in Taiwan, 1997 to 2010. (DOC) [file pone.0061921.s003.doc]

**Table S2. Microbiological, epidemiological, and clinical characteristics and outcomes of *Cryptococcus gattii* in Taiwan, 1997 to 2010**

| No. | Age | Sex | Year | Month | Geographic distribution | Isolate No. | Specimen | Geno type | AMB MIC | FCT MIC | FLU MIC | VOR MIC | Classification | Underlying conditions | Antigen titer ≥1:512 | Intracranial pressure ≥250 cmH_2_O | Neurosurgical intervention | Outcomes |
| --- | --- | --- | --- | --- | --- | --- | --- | --- | --- | --- | --- | --- | --- | --- | --- | --- | --- | --- |
| 1 | 34 | F | 1997 | 9 | Central Taiwan | T315 | CSF | VGII | 0.13 | 2 | 8 | 0.25 | Meningoencephalitis | Diabetes mellitus | Serum, CSF | Yes | Not done | Died at 2 weeks |
| 2 | 29 | F | 1999 | 5 | Central Taiwan | T305 | CSF | VGII | 0.06 | 2 | 0.13 | 0.13 | Meningoencephalitis | No | CSF | No | Not done | Survive |
| 3 | 22 | M | 2004 | 4 | Central Taiwan | T124 | CSF | VGI | 0.25 | 0.5 | 4 | 0.06 | Meningoencephalitis | HIV, Kaposi's sarcoma | CSF | Yes | Done | Survive |
| 4 | 35 | M | 2005 | 8 | Eastern Taiwan | T054 | CSF | VGI | 0.25 | 1 | 1 | 0.03 | Meningoencephalitis | No | CSF | No | Not done | Died at 2 weeks |
| 5 | 43 | M | 2006 | 7 | Southern Taiwan | T169 | CSF | VGII | 0.5 | 2 | 8 | 0.25 | Meningoencephalitis | No | Serum, CSF | Yes | Done | Died at 10 weeks |
| 6 | 68 | M | 2007 | 4 | Central Taiwan | T317 | CSF | VGI | 0.25 | 0.5 | 2 | 0.03 | Meningoencephalitis | CVA | No | Yes | Not done | Survive |
| 7 | 43 | F | 2008 | 3 | Central Taiwan | T107 | CSF | VGII | 0.5 | 1 | 16 | 0.25 | Meningoencephalitis | No | Serum, CSF | Yes | Done | Survive |
| 8 | 32 | F | 2009 | 6 | Northern Taiwan | T174 | CSF | VGII | 0.5 | 2 | 8 | 0.25 | Meningoencephalitis | Transplantation (Kidney) | Serum, CSF | Yes | Not done | Died at 10 weeks |
| 9 | 41 | M | 2010 | 8 | Eastern Taiwan | T234 (T228) | Pleural effusion, (CSF) | VGII | 1 | 1 | 16 | 0.13 | Meningoencephalitis | No | No | No | Not done | Survive |

Abbreviations: M: male, F: female; CSF: cerebrospinal fluid; AMB: amphotericin B, FCT: flucytosine, FLU: fluconazole, VOR: voriconazole, MIC: minimal inhibition concentration (µg/ml); HIV: human immunodeficiency virus; CVA: cerebrovascular accident. A sequential isolate from the same patient was indicated in parentheses.
