# Supplementary material for: Microbiological, Epidemiological, and Clinical Characteristics and Outcomes of Patients with Cryptococcosis in Taiwan, 1997–2010
Source: PLoS One. 2013 Apr 17;8(4):e61921. doi: 10.1371/journal.pone.0061921 (PMC3629109; doi:10.1371/journal.pone.0061921)
Supplement: Table S3 — Microbiological, epidemiological, and clinical characteristics and outcomes of cryptococcosis due to Cryptococcus VNI isolates with antifungal minimum inhibition concentration above epidemiologic cutoff values in Taiwan, 1997 to 2010. (DOC) [file pone.0061921.s004.doc]

**Table S3. Microbiological, epidemiological, and clinical characteristics and outcomes of cryptococcosis due to *Cryptococcus* VNI isolates with antifungal minimum inhibition concentrations above epidemiologic cutoff values in Taiwan, 1997 to 2010.**

| No. | Age | Sex | Year | Month | Isolate No. | Specimen | AMBMIC | FCT MIC | FLU MIC | VOR MIC | Classification | Underlying conditions | Antigen titer ≥1:512 | Intracranial pressure ≥250 cmH_2_O | Neurosurgical intervention | Outcomes |
| --- | --- | --- | --- | --- | --- | --- | --- | --- | --- | --- | --- | --- | --- | --- | --- | --- |
| 1 | 41 | M | 2003 | 7 | T301 | Blood | **1** | 2 | 4 | 0.06 | Meningoencephalitis | HIV | Serum | Yes | No | Died at 2 weeks |
| 2 | 37 | M | 2003 | 10 | T304 | Bone marrow | 0.13 | 2 | **16** | 0.25 | Others (cryptococcemia) | HIV, HBV | Negative | NA | No | Survive |
| 3 | 54 | M | 2006 | 6 | T004 | Blood | 0.5 | **32** | 2 | 0.06 | Others (cryptococcemia) | Kidney disease, CVA | No | NA | No | Survive |
| 4 | 33 | M | 2006 | 8 | T007 | CSF | **1** | 1 | 2 | 0.03 | Meningoencephalitis | No | CSF | Yes | Yes | Survive |
| 5 | 41 | M | 2006 | 8 | T008 | CSF | **1** | 1 | 2 | 0.06 | Meningoencephalitis | HIV, HBV | Serum, CSF | No | No | Survive |
| 6 | 59 | M | 2007 | 6 | T018 | Blood | **1** | 1 | 4 | 0.06 | Meningoencephalitis | Diabetic mellitus, Kidney disease | CSF | No | No | Died at 10 weeks |
| 7 | 51 | F | 2009 | 7 | T175 | Blood | **1** | 1 | 2 | 0.03 | Meningoencephalitis | HBV, Kidney disease | Serum, CSF | No | No | Died at 10 weeks |
| 8 | 33 | M | 2009 | 12 | T195 | Blood | **1** | 4 | 8 | 0.13 | Others (cryptococcemia) | HIV, HBV | Serum | NA | No | Survive |
| 9 | 40 | M | 2010 | 2 | T204 | Lung tumor | **1** | 0.5 | 4 | 0.06 | Meningoencephalitis | HIV, HBV | Serum, CSF | No | No | Survive |

Abbreviations: M: male, F: female, AMB: amphotericin B, FCT: flucytosine, FLU: fluconazole; VOR: voriconazole; MIC: minimal inhibition concentration (µg/ml); HIV: human immunodeficiency virus; HBV: hepatitis B virus; NA: not available. MIC values indicated by underlined bold characters were higher than epidemiologic cutoff values.
